# Human CD57^+ ^germinal center-T cells are the major helpers for GC-B cells and induce class switch recombination

**DOI:** 10.1186/1471-2172-6-3

**Published:** 2005-02-04

**Authors:** Jong R Kim, Hyung W Lim, Seung G Kang, Peter Hillsamer, Chang H Kim

**Affiliations:** 1 Laboratory of Immunology and Hematopoiesis, Department of Pathobiology; Purdue Cancer Center; Bindley Bioscience Center, VPTH Room 126, 725 Harrison St. Purdue University, West Lafayette, IN 47907, USA; 2 Biochemistry and Molecular Biology Program, Purdue University, West Lafayette, IN 47907, USA; 3 Sagamore Surgical Center, Lafayette, IN 47909, USA

## Abstract

**Background:**

The function of CD57^+ ^CD4^+ ^T cells, constituting a major subset of germinal center T (GC-Th) cells in human lymphoid tissues, has been unclear. There have been contradictory reports regarding the B cell helping function of CD57^+ ^GC-Th cells in production of immunoglobulin (Ig). Furthermore, the cytokine and co-stimulation requirement for their helper activity remains largely unknown. To clarify and gain more insight into their function in helping B cells, we systematically investigated the capacity of human tonsil CD57^+ ^GC-Th cells in inducing B cell Ig synthesis.

**Results:**

We demonstrated that CD57^+ ^GC-Th cells are highly efficient in helping B cell production of all four subsets of Ig (IgM, IgG, IgA and IgE) compared to other T-helper cells located in germinal centers or interfollicular areas. CD57^+ ^GC-Th cells were particularly more efficient than other T cells in helping GC-B cells but not naïve B cells. CD57^+ ^GC-Th cells induced the expression of activation-induced cytosine deaminase (AID) and class switch recombination in developing B cells. IgG1-3 and IgA1 were the major Ig isotypes induced by CD57^+ ^GC-Th cells. CD40L, but not IL-4, IL-10 and IFN-γ, was critical in CD57^+ ^GC-Th cell-driven B cell production of Ig. However, IL-10, when added exogenously, significantly enhanced the helper activity of CD57^+ ^GC-Th cells, while TGF-β1 completely and IFN-γ partially suppressed the CD57^+ ^GC-Th cell-driven Ig production.

**Conclusions:**

CD57^+^CD4^+ ^T cells in the germinal centers of human lymphoid tissues are the major T helper cell subset for GC-B cells in Ig synthesis. Their helper activity is consistent with their capacity to induce AID and class switch recombination, and can be regulated by CD40L, IL-4, IL-10 and TGF-β.

## Background

In germinal centers (GC), B cells undergo clonal expansion, somatic hyper-mutation in the variable region of antibody genes [1-3] and class switch recombination (CSR) from IgM to IgG, IgA, and IgE [4-8], processes that are dependent on helper T cells [9-11]. Antibodies to the CD57 epitope (HNK-1) have been used to identify a T cell type in germinal centers in human tonsils, spleen and lymph nodes. These cells are CD4^+ ^T cells [12-14], exhibit a memory phenotype (CD45RO^+^CD45RA^-^) [15] and are not cytolytic [16]. CD57^+ ^GC-Th cells proliferate only when they are TCR-activated in the presence of IL-2 [17,18]. CD57^+ ^GC-Th cells express the B-cell zone homing chemokine receptor CXCR5 but not the T cell zone homing chemokine receptor CCR7, a pattern consistent with their specific localization in GC [19]. Based upon their non-polarized cytokine profile, localization in GC and potential helper activity, it has been proposed that CD57^+ ^GC-Th cells may constitute a novel effector T cell subset distinct from other well known effector T cell subsets such as Th1 and Th2 cells [20]. Using a gene expression profiling study, we determined that CD57^+ ^GC-Th cells are remotely related to other memory/effector T cells in global gene expression [21]. The microarray study also revealed that CD57^+ ^GC-Th cells have the unique capacity to produce CXCL13, a follicle chemokine implicated in recruitment of CXCR5^+ ^cells [22,23] and development of follicles/GCs [24]. Because of their specific localization in germinal centers, the activities of CD57^+ ^GC-Th cells on B cell proliferation and antibody production have been studied by several groups of scientists [19,25-27]. The results of these previous studies reveled unique features of CD57^+ ^GC-Th cells, but, when combined, they are inconclusive and widely vary from negative to neutral or positive in assessing the helper activities of CD57^+ ^GC-Th cells.

To clarify and gain more insight into their function in helping B cells, we systematically investigated the capacity of human tonsil CD57^+ ^GC-Th cells in inducing B cell Ig synthesis in naïve vs. germinal center B (GC-B) cells in comparison with other T cell subsets in human tonsils. We show that CD57^+ ^GC-Th cells are more efficient than other germinal center or interfollicular T cells in supporting B cell production of Ig. CD57^+ ^GC-Th cells, when compared to other T cells, have better helper activity for GC-B cells than for naïve B cells. CD57^+ ^GC-Th cells induced the expression of activation-induced cytosine deaminase (AID) and CSR in developing B cells. CD40L, but not other major cytokines, is critical for the helper activity of CD57^+ ^GC-Th cells. IL-10 positively and TGF-β1 negatively regulate the helper activity of CD57^+ ^GC-Th cells.

## Results

### Distribution and identification of T helper cell subsets in tonsils

**Figure 1:**
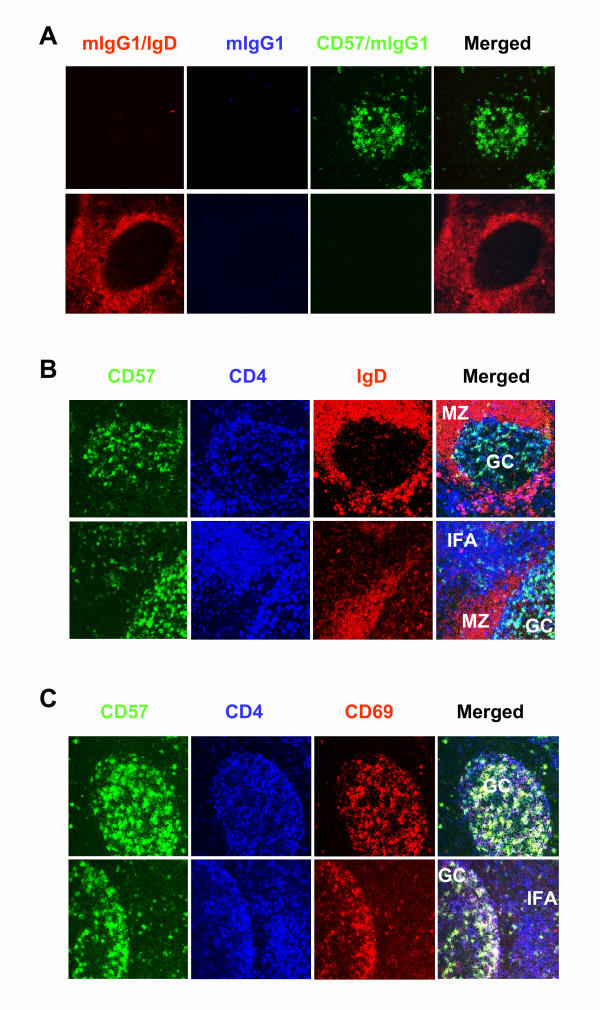
Distribution of CD4^+ ^T helper cells in tonsils. Frozen tonsil sections were stained with anti-IgD (PE, red) or anti-CD57 (FITC, green) and isotype control antibodies in panel group A to show the background staining of the system. In panel group B, sections were stained with anti-CD57 (FITC), anti-IgD (PE) and anti-CD4 (APC). In panel group C, sections were stained with anti-CD57 (FITC), anti-CD69 (PE) and anti-CD4 (APC). Two different sections were shown in each group of panels. Stained sections were analyzed with a confocal microscope. GC-Th cells can be divided into CD57^+ ^and CD57^- ^T cells, both of which are CD69^+^. A few CD69^+ ^or CD57^+ ^T cells are found outside of GC. Most CD4^+ ^T cells in the interfollicular areas (IFA or T cell-rich zone) are CD57^- ^and CD69^-^. GCs are surrounded by the ring of mantle zones (MZ) filled with IgD^+ ^cells. A representative set of images from three different specimens are shown.

We examined the distribution of T helper cell subsets in human tonsils based upon the expression of CD4, CD57 and CD69. As reported previously [12,19,28,29], most CD57^+ ^CD4^+ ^T cells are located in germinal centers surrounded by IgD^+ ^naïve B cells (Fig. 1). Small numbers of CD57^+ ^T cells were also present in the interfollicular areas (IFA or T cell-rich zone) surrounding GC. Although some are found in IFA, CD69^+ ^CD4^+ ^T cells were also preferentially found in GC (Figure 1C). In contrast, the T cells in IFA were mostly negative for CD69 expression. Therefore, CD57, CD69 and CD4 are useful markers to identify CD57^+ ^GC-Th cells and other T cell subsets differentially localized in tonsils: CD4^+^CD57^+ ^cells (mainly in GC), CD4^+^CD57^-^CD69^+ ^cells (mainly in GC and a minor proportion in IFA), and CD4^+^CD57^-^CD69^- ^cells (mainly in IFA).

### CD57^+ ^GC-Th cells are highly efficient in supporting Ig production by B cells

**Figure 2:**
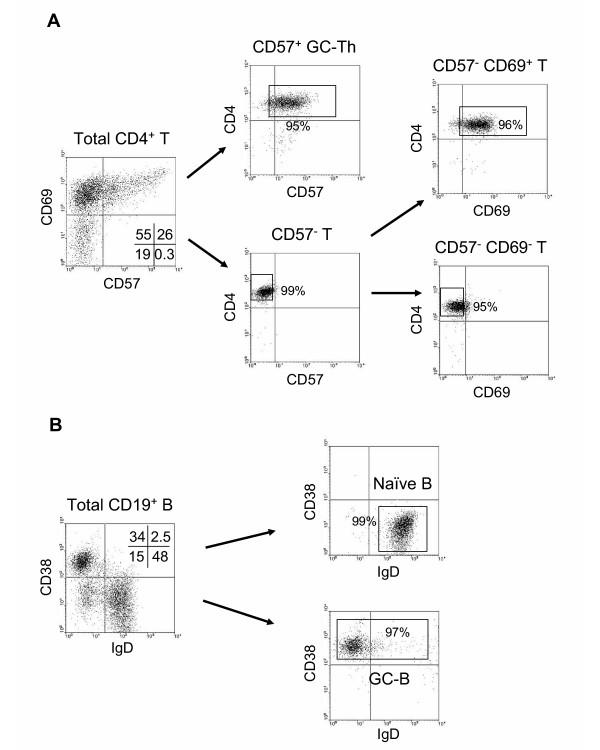
Isolation of human tonsil T cell and B cell subsets examined in this study. T cell subsets and B cell subsets were isolated from tonsils as described in the materials and methods section and were used in this study. The frequencies of the populations in total tonsil CD4^+ ^T or CD19^+ ^B population are 15–25% for CD4^+^CD57^+ ^GC-Th cells, 50–60% for CD57^-^CD69^+ ^T cells, 20–30% for CD57^-^CD69^- ^T cells, 40–60% for CD19^+^CD38^-^IgD^+ ^naïve B cells and 30–40% for CD19^+^CD38^+^IgD^- ^GC-B cells.

**Figure 3:**
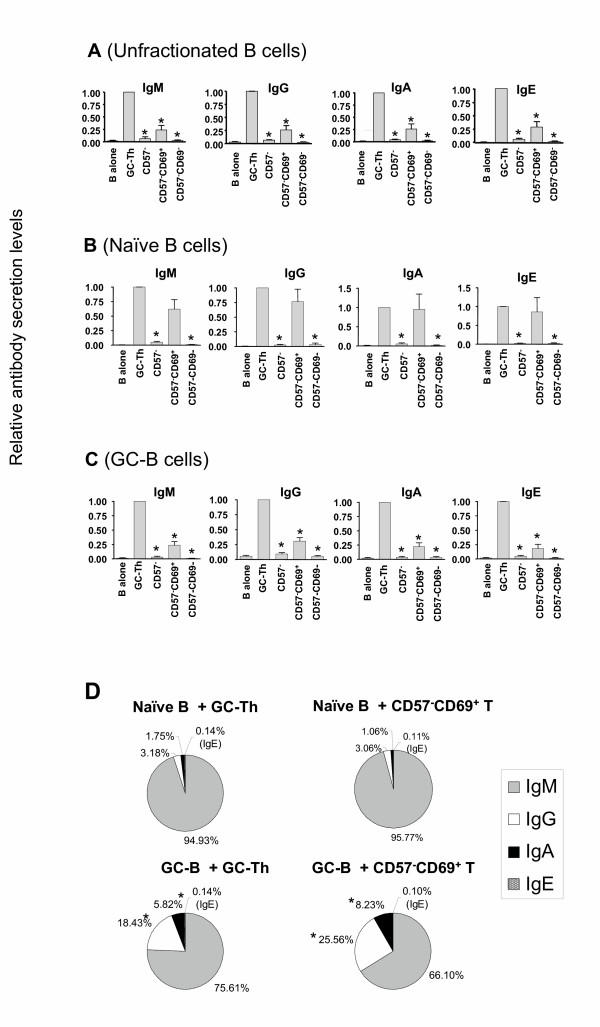
CD57^+ ^GC-Th cells are more efficient than other tonsil CD4^+ ^T cell subsets in helping B cells. (A) CD4^+ ^T cell subsets were cultured with total tonsil CD19^+ ^B cells for 7 days in the presence of SEB. Naïve B cells (C) or GC-B cells (D) were cultured with equal numbers of CD57^+^GC-Th cells or other T cell subsets (CD57^-^, CD57^-^CD69^+ ^and CD57^-^CD69^- ^T cells) for 7 days followed by ELISA for IgM, IgG, IgA and IgE. Data from 5 independent experiments were combined and the averages are shown with standard errors. Relative production levels to CD57^+ ^GC-Th cells are shown. *Significant differences from CD57^+ ^GC-Th cells. The absolute Ig production levels (ng/ml) in panel A (GC-Th + Total B cells) were 5737 ± 1764 (IgM), 2111 ± 1185 (IgG), 577 ± 186 (IgA), and 4.8 ± 2.1 (IgE). The absolute Ig production levels (ng/ml) in panel B (GC-Th cells + naïve B cells) were 2045 ± 697 (IgM), 63 ± 21 (IgG), 40 ± 23 (IgA), and 2.9 ± 1.2 (IgE). The average levels (ng/ml) of Ig produced in the cultures of GC-Th cells and GC B cells were 750 ± 279 (IgM), 175 ± 52 (IgG), 51 ± 13 (IgA), and 1.0 ± 0.5 (IgE). (D) Isotype composition of the Ig induced by CD57^+ ^GC-Th cells. Naïve B cells or GC-B cells were cultured with equal numbers of CD57^+^GC-Th cells or CD57^-^CD69^+ ^T cells for 7 days followed by ELISA for IgM, IgG, IgA and IgE. Data from 4 independent experiments were combined and the averages are shown with standard errors. *Significant differences between naïve and GC-B cells, but not between the two T cell subsets, were observed.

Based upon the information obtained in Figure 1, the total tonsil CD4^+ ^T cell population was fractionated into CD57^+ ^GC-Th cells (all of these cells are CD69^+^), total CD57^- ^T cells, CD57^-^CD69^+ ^T cells and CD57^-^CD69^- ^T cells (Figure 2). We compared the B cell helping activity of CD57^+ ^GC-Th cells with that of other CD4^+ ^T cell subsets. We co-cultured each of the isolated T cell subsets with syngeneic tonsil CD19^+ ^B cells in the presence of SEB, a superantigen that conjugates MHC class II molecules and TCR (Figure 3). B cell receptors were cross-linked by Ab to human Ig μ chain and human Ig (H + L) chain prior to culture to provide BCR activation signals mimicking the antigen signals in vivo. CD57^+ ^GC-Th cells were most efficient in inducing B cell production of IgM, IgG, IgA and IgE among the T cell subsets examined. CD57^- ^CD69^+ ^T cells, many of which are located in GC in a manner similar to CD57^+ ^GC-Th cells, were able to induce the production of antibodies but at significantly lower levels compared to CD57^+ ^GC-Th cells (Figure 3A). T cell stimulation, in this study by SEB, was required for efficient induction of the B cell helper activity as it enhanced Ig production up to ~1000 (not shown).

### GC-B cells are the preferred target cells for the helper activity of CD57^+ ^GC-Th cells

Because of their specific localization in germinal centers, the physiological target cells for CD57^+ ^GC-Th cells would be GC-B cells rather than naïve B cells. We compared the helper activities of CD57^+ ^GC-Th cells and CD57^- ^CD69^+/- ^CD4^+ ^T cell subsets for B cells. In this study, we fractionated CD19^+ ^B cells into two groups: IgD^+^CD38^- ^naïve B and CD38^+ ^IgD^+/- ^GC B cells as shown in Figure 2B. CD57^+ ^GC-Th cells, when co-cultured with GC-B cells, were significantly more efficient than CD57^-^CD69^+ ^T cells in inducing the production of all four isotypes of Ig (Figure 3C). However, when co-cultured with naive B cells, CD57^+ ^GC-Th cells were not significantly different from CD57^-^CD69^+ ^T cells in their induction capacity of Ig (Figure 3B). Again, the helper activities of total CD57^- ^T cells and CD57^-^CD69^- ^T cells for naïve and GC-B cells were very low.

The relative composition of IgM, IgG, IgA and IgE produced in response to CD57^+ ^GC-Th cells in the cultures with GC-B vs. naïve B cells was determined. CD57^+ ^GC-Th cells drove the production of IgM, IgG, IgA and IgE in descending order (Figure 3D). Class-switched Ig isotypes such as IgG and IgA were more produced in GC-B cell cultures than in naïve B cell cultures. There was no statistically significant difference between the two T cell subsets (CD57^+ ^GC-Th cells and CD4^+^CD57^-^CD69^+ ^T cells) in the composition of Ig that they induced.

### CD57^+ ^GC-Th cells induce AID expression and class switch recombination in B cells

**Figure 4:**
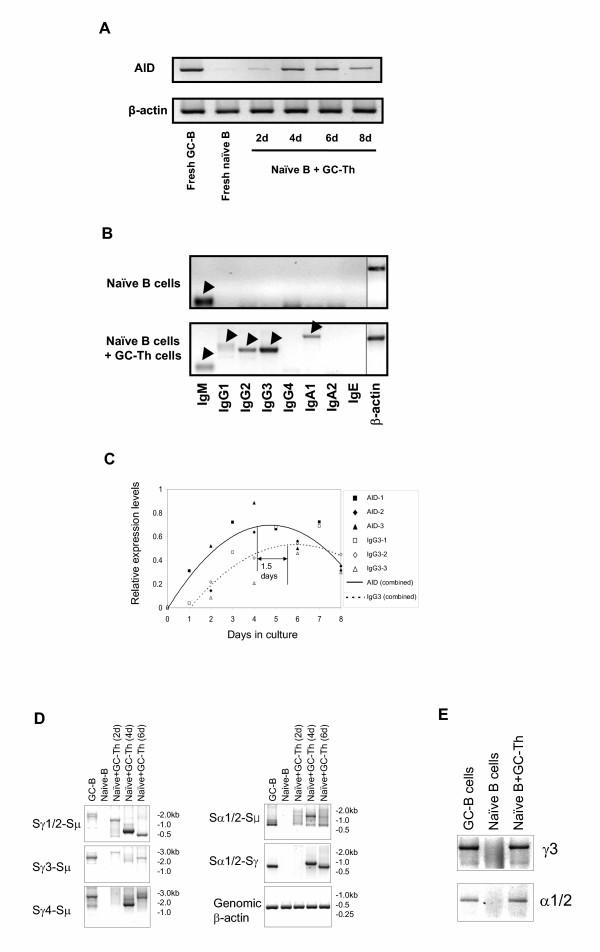
CD57^+ ^GC-Th cells have the capacities to induce AID expression and to support CSR in B cells. IgD^+^CD38^- ^naïve B cells were cultured with CD57^+ ^GC-Th cells for indicated time periods followed by RT-PCR analysis for (A) AID expression and (B) CSR. The sizes of specific PCR products are 152 bp (IgM); 416 bp (IgG1, G2, G3), 904 bp (IgG4); 904 bp (IgA1); 891 bp (IgA2); and 179 bp (IgE). Shown are productive recombination products. (C) The expression kinetics of AID and productive IgG3 transcripts over an 8 day period are shown together in a graph. In this panel, normalized expression levels calculated after dividing the levels of AID amplification by β-actin levels are shown. The time gap to reach the peak levels of the expression between AID and productive IgG3 transcripts is shown by an arrow. Representative data from at least three independent experiments are shown (panels A and B). (D) Identification of extrachromosomal reciprocal DNA recombination products. Naïve B cells were cultured with CD57^+ ^GC-Th cells for indicated time periods and were processed to isolate genomic DNA. Fresh GC-B cells were examined for positive controls. The switch circles were detected by a nested PCR method. Representative data out of three independent experiments are shown. (E) Detection of switch circles by a DC-PCR technique. Naive B cells, CD38^+ ^GC-B cells and naïve B cells cultured with GC-Th cells for 5 days were examined for the presence of γ3 and α1/2 switch circles.

AID expression in the maturating B cells in GC is necessary for CSR and somatic hypermutation. We examined whether CD57^+ ^GC-Th cells have the capacity to induce AID in B cells. Naïve B cells were co-cultured with CD57^+ ^GC-Th cells, and AID expression was examined (Figure 4A). CD57^+ ^GC-Th cells induced AID in activated B cells with peaks around days 3–4. CD57^+ ^GC-Th cells were able to induce the expression of productive V_H_DJ_H_-C_H _Ig transcripts. The major subtypes of Ig transcripts in response to CD57^+ ^GC-Th cells were IgG3, IgG2, IgG1 and IgA1 (Figure 4B). When the peak expression levels of AID and the productive V_H_DJ_H_-C_H _IgG3 transcript (the most readily detected Ig transcript) were compared, AID expression preceded the expression of IgG3 transcript by 1–2 days in culture (Figure 4C).

Ig class switch recombination between tandemly repeated S regions located 5' to each C_H _gene generates switch circles. We used a nested PCR technique designed to specifically detect switch circles but not genomic Ig sequences. Freshly isolated GC-B, but not naïve B cells, contained switch circles, which were detected as smeared multiple bands on agarose gels as expected. Naïve B cells cultured with CD57^+ ^GC-Th cells generated detectable switch circles in a time-dependent manner (Figure 4D). We also used a DC-PCR technique [30] to detect γ3 and α1/2 switch circles (Figure 4E). Again, GC-Th cells induced switch circles in the naïve B cells cultured with GC-Th cells.

### CD40L signal is necessary for, while cytokines modulate, the helper activity of CD57^+ ^GC-Th cells

**Figure 5:**
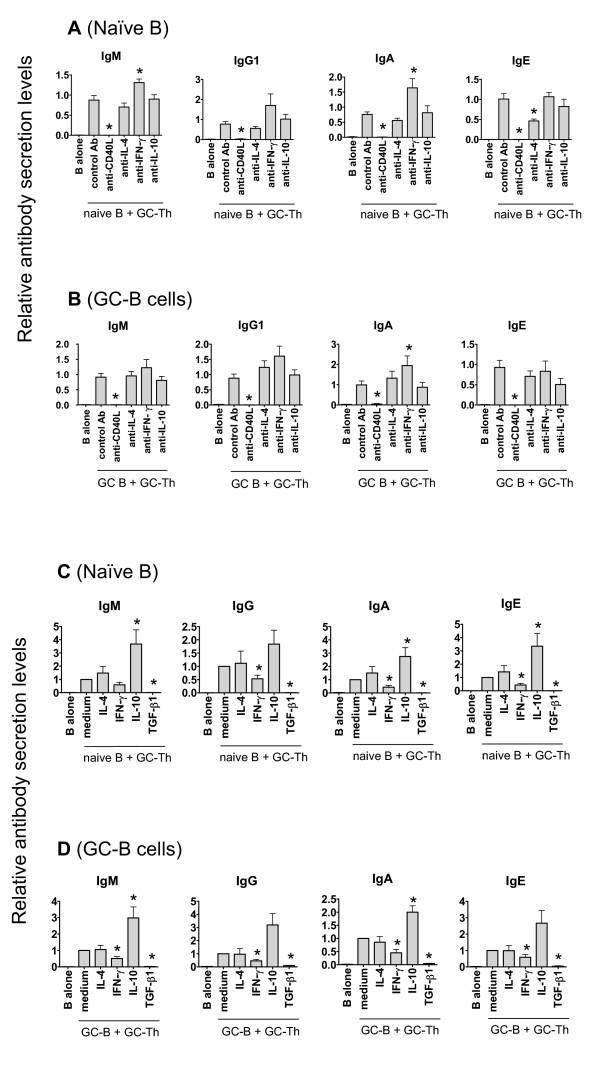
CD40L and cytokines in regulation of the helper activity of CD57^+ ^GC-Th cells. (A and B) Effects of endogenous CD40L and cytokines on the helper activity of CD57^+ ^GC-Th cells were determined. In cultures of CD57^+ ^GC-Th cells with naïve or GC-B cells, neutralizing antibodies to IL-4, IL-10, IFN-γ or CD40L or control antibodies (mouse IgG1) were added. *Significant differences from the control group (control antibody). (C and D) Effects of exogenously added cytokines on the helper activity of CD57^+ ^GC-Th cells were determined. To cultures of CD57^+ ^GC-Th cells with naïve or GC-B cells, IL-4, IL-10, IFN-γ and TGF-β1 were added separately. Cells were cultured for 7 days followed by ELISA for IgM, IgG, IgA and IgE. Relative Ig secretion levels (the medium control = 1) obtained from 9 independent experiments were combined, and averages and standard errors are shown. *Significant differences from the control group (medium).

Cytokines and CD40L regulate B cell maturation and Ig production. We examined whether CD40L, IL-4, IL-10 and IFN-γ play any roles in the CD57^+ ^GC-Th cell-driven B cell production of Ig. In cultures with naïve B cells, the blockage of CD40L by neutralizing antibody completely suppressed the helper activity of CD57^+ ^GC-Th cells in inducing the B cell production of IgM, IgG1, IgA and IgE (Figure 5A). In this case, IgG1 was measured instead of total IgG to avoid cross-reaction of the polyclonal capturing antibody for IgG with the neutralizing antibodies to cytokines. Blockage of IL-4 partially but specifically suppressed the production of IgE, but it did not significantly suppressed other isotypes. In contrast, blockage of IFN-γ enhanced the production of IgM, IgG1 and IgA but not IgE. Since CD40L is essential for the helper activity of CD57^+ ^GC-Th cells, we examined CD57^+ ^GC-Th cells and other T cells for the expression of surface CD40L. Freshly isolated CD57^+ ^GC-Th cells expressed CD40L, which became up-regulated upon T cell activation within hours (data not shown), whereas CD4^+^CD57^-^CD69^- ^interfollicular T cells did not express CD40L at significant levels. There was no significant difference in the expression of surface CD40L between CD57^+ ^and CD57^- ^CD69^+ ^cells.

In the cultures with GC-B cells, blocking of CD40L again completely suppressed the B cell helping activity of CD57^+ ^GC-Th cells (Figure 5B). However, IL-4 neutralization did not significantly affect the IgE production induced by CD57^+ ^GC-Th cells, an activity different from that for naïve B cells. For GC-B cells, IFN-γ neutralization significantly increased the production of IgA as it did for naive B cells. The effects of IFN-γ neutralization on other Ig isotypes were smaller. While a slight decrease of IgE production in the cultures of GC-B cells and CD57^+ ^GC-Th cells was observed, neutralization of endogenous IL-10 did not have any statistically significant effect on CD57^+ ^GC-Th cell-driven Ig production in the cultures of either naïve or GC-B cells.

### Exogenously-added IL-10 enhances while TGF-β1 completely suppresses the B cell helping activity of CD57^+ ^GC-Th cells

To further examine the regulatory effect of cytokines, IL-4, IL-10, IFN-γ and TGF-β1 were exogenously added to the cultures of CD57^+ ^GC-Th cells with B cells (Figure 5C and 5D). In cultures of CD57^+ ^GC-Th cells with naïve B cells, exogenously added IL-4 enhanced the production of some subsets of Ig, but this effect was small and not statistically significant (Figure 5C). However exogenously added IFN-γ significantly suppressed the production of IgG, IgA and IgE. IL-10, when added exogenously, was highly efficient in enhancing the production of the four subsets of Ig. TGF-β1 completely suppressed the B cell-helping capacity of CD57^+ ^GC-Th cells for naive B cells.

In cultures of CD57^+ ^GC-Th cells with GC-B cells, IL-10 was again highly effective in enhancing the helper activity of CD57^+ ^GC-Th cells, while TGF-β1 completely suppressed it (Figure 5D). IFN-γ partially but significantly suppressed the production of IgM, IgG, IgA and IgE. Exogenous IL-4 added to the cultures had no effect on the CD57^+ ^GC-Th cell-driven Ig production in this condition (Figure 5D), which is in line with the negligible effect of anti-IL-4 on GC-B cells in Figure 5B.

## Discussion

CD57^+ ^GC-Th cells are unique CD4^+ ^T cells. They express the follicle homing receptor CXCR5 but lack the T cell area localization receptor CCR7 [19], and reside specifically in germinal centers [12-14]. CD57^+ ^GC-Th cells proliferate only when appropriate signals such as TCR, CD28 and IL-2 are provided [17,18]. GC-Th cells are widely disseminated and diverse in their TCR sequence [31]. CD57^+ ^GC-Th cells can express CD40L, ICOS and CXCL13 but are non-polarized T cells in their cytokine profile [21]. It has been controversial and unclear whether CD57^+ ^GC-Th cells are intrinsically more efficient in helping B cells than other T cells or they are simply localized in germinal centers without any significant differences from other T cells in their capacity as helpers. In this report, we systematically investigated the effector function of CD57^+ ^GC-Th cells in regulation of B cell immunoglobulin production and its regulation.

When compared for their helper activities in inducing Ig synthesis by total B cells, CD57^+ ^GC-Th cells were most efficient among the T cell subsets in tonsils. CD57^+ ^GC-Th cells were particularly more efficient in their helper activity for GC-B cells vs. naïve B cells. CD57^-^CD69^+ ^T cells were equally efficient to CD57^+ ^GC-Th cells in inducing naïve B cell differentiation for Ig production, while they were less effective than CD57^+ ^GC-Th cells in helping GC-B cells. This preference of CD57^+ ^GC-Th cells for GC-B cells is physiologically relevant, since both the helper T cell subset and target B cells are specifically present in germinal centers. Therefore, CD57^+ ^GC-Th cells would constitute an ideal T helper subset that can drive GC-B cell differentiation in germinal centers.

The effects of cytokines such as IL-4, IL-10, IFN-γ and CD40L on B cells in humans and mice have been well documented. It is considered that CD40L is a critical factor [4,11,32-37], and IL-4 and IL-10 are positive factors in regulation of B cell Ig production [38-44]. IFN-γ induces class switch to certain isotypes while it inhibits to others [45,46]. In this study of the helper activity of CD57^+ ^GC-Th cells, the positive role of IL-4 in promoting Ig production was valid only for IgE, but not IgG and IgA in the cultures of naïve B cells with CD57^+ ^GC-Th cells (Figure 5). GC-B cells were even more resistant to the neutralization of IL-4 than naïve B cells in CD57^+ ^GC-Th-cell driven Ig production. This smaller than expected effect of IL-4 may be due to the fact that there is not much IL-4 to neutralize in the cultures of GC-Th cells. This also suggests that GC-Th cells may provide helper signals to GC-B cells that are not significantly affected by IL-4.

AID [47] is a molecule essential for somatic hypermutation, CSR and Ig gene conversion [48-54]. We showed in this study that CD57^+ ^GC-Th cells can induce AID expression (Figure 4A). This capacity is consistent with their ability to induce class switch recombination, which can be detected within a few days in the cultures of naïve B cells with CD57^+ ^GC-Th cells. CD57^+ ^GC-Th cells can induce the expression of productive IgG1-3 and IgA1 transcripts. However, CD57^+ ^GC-Th cells were inefficient in induction of IgE (Figure 3, 4 and 5), which is consistent with their poor production capacity of IL-4 [19].

CD40L appears to be essential for the helper activity of CD57^+ ^GC-Th cells. CD40L was required for the synthesis of all Ig isotypes in all the conditions tested regardless of whether the target B cells for CD57^+ ^GC-Th cells were naïve or GC-B cells. While neutralization of IL-10 did not have any significant effect on the CD57^+ ^GC-Th cell-driven Ig synthesis, exogenous IL-10 was highly effective in enhancing the Ig synthesis in our study. This could be due to insufficient neutralization of the IL-10 produced by CD57^+ ^GC-Th cells, which are known to produce IL-10 upon TCR activation [19]. Another possibility is that higher concentration of IL-10 than the level produced by CD57^+ ^GC-Th cells may be necessary to significantly enhance the Ig response. Exogenous IFN-γ negatively regulates the CD57^+ ^GC-Th cell-driven Ig synthesis, suggesting the potential roles of Th1 cells or other IFN-γ producing cells in regulation of the CD57^+ ^GC-Th cells' helper activity. TGF-β1 plays dual roles: it is a switch factor for IgA and a potent immunosuppressive cytokine that inhibits Ig synthesis [55]. We did not detect any switching effect but were able to detect its suppressive activity for the CD57^+ ^GC-Th cell response. This could be due to the fact that the culture conditions (e.g. the saturating concentration of TGF-β) employed in our study appear to favor the detection of the suppressive function of TGF-β. Taken together, these results imply that Th1, Th2 and regulatory T cells, if present in germinal centers, could positively or negatively control the function of CD57^+ ^GC-Th cells in regulation of humoral immune responses. Indeed, there are regulatory T cells in GCs that express surface TGF-β and can effectively suppress the function of CD57^+ ^GC-Th cells [56].

## Conclusions

Our results demonstrated the capacity of CD57^+ ^GC-Th cells in supporting CSR and Ig synthesis in B cells, and revealed the factors that regulate their activity, thereby substantiating the so-far inconclusive function of CD57^+ ^GC-Th cells. The fact that these T cells have preferential and efficient helper activity for GC-B cells and are specifically localized in GCs in large numbers suggests that CD57^+ ^GC-Th cells are probably the major T helper subset responsible for supporting B cell differentiation for Ig production in germinal centers.

## Methods

### Cell isolation

Mononuclear cells were prepared by density gradient centrifuge on histopaque 1077 (Sigma-Aldrich, St. Louis) from human tonsil pathological specimens obtained from young patients (3–10 yr) undergoing tonsillectomy to relieve obstruction of respiratory passages and improve drainage of the middle ear at Sagamore Surgical Center (Lafayette, IN). The use of human pathological specimens in this study was approved by the institutional review board at Purdue University. CD4^+ ^T cells (purity >97%) were isolated by depleting non-CD4^+ ^T cells using a magnetic bead depletion method (Miltenyi Biotec, Auburn, CA). After staining of the isolated CD4^+ ^T cells with appropriate antibodies, CD57^+ ^GC-Th cells (purity >95%) were isolated by a positive magnetic selection method (Miltenyi Biotec). CD4^+^CD57^-^CD69^+ ^and CD4^+^CD57^-^CD69^- ^T cell subsets (purity >95%) were further isolated from the CD57^- ^T cell fraction by magnetically selecting CD69^+ ^T cells (Miltenyi Biotec). Total B cells were isolated by rosetting with 2-amino-ethylisothiouronium bromide (AET)-treated sheep red blood cells followed by CD4^+ ^T cell depletion (CD19^+ ^cells > 99.5%). Naïve B cells (CD19^+^IgD^+ ^cells >99%) were isolated from the total B cell fraction by depleting CD38^+ ^T cells followed by positive magnetic selection of IgD^+ ^B cells. CD19^+^CD38^+^IgD^+/- ^GC-B cells (purity >95%) were isolated from the tonsil CD19^+ ^B cells as described before [57] using anti-CD44, anti-IgD antibodies and pan-mouse IgG beads (Dynal, Brown Deer, WI).

### Cell culture

All cell cultures were performed in RPMI1640 medium supplemented with 10% FBS, gentamycin, streptomycin, and penicillin. To cross-link the B cell receptors, isolated B cells were incubated for 2 h at 4°C with Sepharose-conjugated rabbit Ab to human Ig μ chain and human Ig (H + L) chain (Irvine Scientific, Santa Ana, CA; mixed 1:1 at 2 μg/ml), and then washed with cold PBS. 10^5 ^T and 10^5 ^B cells were co-cultured, unless indicated otherwise, in each well of 48-well plates in the presence of Staphylococcal enterotoxin B (SEB; 1 μg/ml, Sigma-Aldrich, St. Louis, MO). Cells were incubated in 5% CO^2 ^incubators at 37°C for 3–8 days. Recombinant IL-4, IL-10, and TGF-β1 were purchased from R&D systems (Minneapolis, MN). Recombinant IFN-γ was obtained from BD Pharmingen (San Diego, CA). Purified CD154-blocking antibody (24–31) was obtained from Ancell Corporation (Bayport, MN). IL-4-blocking antibody (MP4-25D2) was purchased from BD Pharmingen. Blocking antibodies for IFN-γ (25718.111) and IL-10 (23738.111), and IgG1 isotype control antibody (11711.11) were purchased from R&D systems. All antibodies and reagents added to culture were azide-free. Cytokines were added at saturating concentrations: IL-4 (40 ng/ml), IL-10 (40 ng/ml), IFN-γ (200 ng/ml) and TGF-β1 (10 ng/ml). Neutralizing antibodies were added at following concentrations: anti-CD40L (20 μg/ml), anti-IL-4 (5 μg/ml), anti-IL-10 (5 μg/ml), anti-IFN-γ (2.5 μg/ml) and isotype antibody (5 μg/ml).

### Flow cytometry analysis

T cells were stained with anti-human CD57 (NK-1; FITC, BD Pharmingen), anti-human CD69 (FN-50; FITC, BD Pharmingen), anti-human CD4 (S3.5; R-PE, Caltag Laboratories, Burlingame, CA), and anti-human CD3 (UCHT1; APC, BioLegend, San Diego, CA). B cells were stained with anti-CD19 (4G7; PerCP, BD Pharmingen), anti-human IgD (IAb-2, FITC, BD Pharmingen), anti-human CD38 (HTT2; R-PE, BD Pharmingen), and anti-human CD3 (UCHT1; APC, BioLegend). Stained cells were analyzed using a 4-color FACSCalibur™ (BD Biosciences).

### In situ fluorescent immunohistochemistry

Frozen sections of tonsils were acetone-fixed and stained using antibodies to CD57 (BD Biosciences – Pharmingen; clone NK-1, labeled with FITC), CD69 (BD Biosciences – Pharmingen; clone FN50, labeled with FITC), IgD (BD Biosciences – Pharmingen; clone IA6-2, labeled with PE) and/or CD4 (Caltag Laboratories; clone S3.5, labeled with APC). Stained sections were analyzed using a confocal microscopy system (Bio-Rad MRC 1024UV and Nikon Diaphot 300 microscope) at Purdue Cytometry Lab.

### ELISA

Culture supernatants were assayed by ELISA as previously described [19]. The sensitivity of this ELISA system is greater than 5 ng/ml, 300 pg/ml, 30 pg/ml, 600 pg/ml, and 15 pg/ml for IgM, IgG, IgG1, IgA and IgE, respectively.

### Detection of productive V_H_DJ_H_-C_H _Ig transcripts and reciprocal DNA recombination products

Total RNA was extracted from cultured cells with Trizol reagent (Invitrogen, Carlsbad, CA), and was reverse-transcribed into cDNAs with SuperScript™ First-Strand Synthesis System for RT-PCR (Invitrogen) according to the manufacturer's protocol. The primer pairs used in this study were designed by Cerutti et al. [37]: IgM, FR3 forward (5'-GAC ACG GCT GTG TAT TAC TGT GCG-3') and Cμ reverse (5'-CCG AAT TCA GAC GAG GGG GAA AAG GGT T-3'); IgG1, FR3 forward and Cγ1 reverse (5'-GTT TTG TCA CAA GAT TTG GGC TC-3'); IgG2, FR3 forward and Cγ2 reverse (5'-GTG GGC ACT CGA CAC AAC ATT TGC G-3'); IgG3, FR3 forward and Cγ3 reverse (5'-TTG TGT CAC CAA GTG GGG TTT TGA GC-3'); IgG4, FR3 forward and Cγ4 reverse (5'-ATG GGC ATG GGG GAC CAT TTG GA-3'); IgA1, FR3 forward and Cα1 reverse (5'-GGG TGG CGG TTA GCG GGG TCT TGG-3'); IgA2, FR3 forward and Cα2 reverse (5'-TGT TGG CGG TTA GTG GGG TCT TGC A-3'); IgE, FR3 forward and Cε reverse (5'-CGG AGG TGG CAT TGG AGG-3'); human β-actin, actin forward (5'-ATG TTT GAG ACC TTC AAC AC-3') and actin reverse (5'-CAC GTC ACA CTT CAT GAT GG-3'). PCR reactions were performed on serially diluted cDNA samples using an Eppendorf master cycler (denaturation at 95°C for 15 s, annealing at 55°C for 45 s and extension at 72°C for 30°C; 30–35 cycles). Extrachromosomal switch circles were detected by a nested PCR strategy as previously described by others [37]. Briefly, genomic/extrachromosomal DNA was isolated from fresh or cultured B cells using a QIAamp DNA Mini Kit (Qiagen, Valencia, CA) and was used as templates for amplification of Sγ1-Sμ, Sγ2-Sμ, Sγ3-Sμ, Sγ4-Sμ and Sα-Sγ. The PCR products were subject to second PCR using internal forward 5' Sγ or 5'Iα1/2i and reverse 3'Sμi or 3'γi primer pairs. This method has been verified for specificity using positive controls [37]. Additionally, we amplified genomic β-actin gene as a control using 5'-GTA CCA CTG GCA TCG TGA TGG ACT-3' (G-actin-forward-1 primer) and 5'-ATC CAC ACG GAG TAC TTG CGC TCA-3' (G-actin-reverse-1) for the first PCR; and 5'-AGA AGA GCT ACG AGC TGC CTG AC-3' (G-actin-forward-2) and 5'-TGA GGA CCC TGG ATG TGA CAG CT-3' (G-actin-reverse-2) for the second PCR. Additionally, we used a DC-PCR technique [30,58] to demonstrate the presence of switch circles (γ3 and α1/2) in human B cells. Please see the reference [30] for primer sequences.

### RT-PCR analysis for AID expression

Total RNA was extracted from freshly isolated or cultured cells using Trizol reagent (Invitrogen, Carlsbad, CA), and was reverse-transcribed into cDNAs with SuperScript™ II Reverse Transcriptase. RT-PCR amplification of AID was performed using the two primers: AID-forward (5'-GAT GAA CCG GAG GAA GTT TC-3') and AID-reverse (5'-TCA GCC TTG CGG TCC TCA CAG-3'), which generated a specific 351 bp PCR product after 30 cycles of PCR reaction (30 s at 94°C, 30 s at 60°C, and 60 s at 72°C). β-actin was also amplified as a control.

### Statistical analysis

Student's paired t-test was used. P values smaller than 0.05 were considered significant.

## List of abbreviations used

GC, germinal center; GC-Th cells, germinal center T helper cells; GC-B cells, germinal center B cells; AID, activation-induced cytosine deaminase; CSR, class switch recombination; SEB, staphylococcal enterotoxin B.

## Authors' contributions

CHK conceived, coordinated the study, analyzed the results and wrote the text. JRK, HWL and SGK participated in experiments, data analysis, making figures and proofreading the manuscript. PH provided specimens and helped perform the study. All authors read and approved the manuscript.
